# Microglia dynamics in adolescent traumatic brain injury

**DOI:** 10.1186/s12974-020-01994-z

**Published:** 2020-10-29

**Authors:** Eric Eyolfson, Asher Khan, Richelle Mychasiuk, Alexander W. Lohman

**Affiliations:** 1 Department of Psychology, University of Calgary, 2500 University Drive NW, Calgary, AB T2N1N4 Canada; 2 Hotchkiss Brain Institute, University of Calgary, 3330 Hospital Drive NW, Calgary, AB T2N4N1 Canada; 3 Alberta Children’s Hospital Research Institute, University of Calgary, 3330 Hospital Drive, NW, Calgary, AB T2N4N1 Canada; 4 Department of Neuroscience, Monash University, 6th Floor, The Alfred Centre, 99 Commercial Road, Melbourne, VIC 3004 Australia; 5 Department of Cell Biology and Anatomy, University of Calgary, 3330 Hospital Drive NW, Calgary, AB T2N4N1 Canada

**Keywords:** Synaptic pruning, Glia, Pathophysiology, White matter, Brain maturation, Complement cascade

## Abstract

Repetitive, mild traumatic brain injuries (RmTBIs) are increasingly common in adolescents and encompass one of the largest neurological health concerns in the world. Adolescence is a critical period for brain development where RmTBIs can substantially impact neurodevelopmental trajectories and life-long neurological health. Our current understanding of RmTBI pathophysiology suggests key roles for neuroinflammation in negatively regulating neural health and function. Microglia, the brain’s resident immune population, play important roles in brain development by regulating neuronal number, and synapse formation and elimination. In response to injury, microglia activate to inflammatory phenotypes that may detract from these normal homeostatic, physiological, and developmental roles. To date, however, little is known regarding the impact of RmTBIs on microglia function during adolescent brain development. This review details key concepts surrounding RmTBI pathophysiology, adolescent brain development, and microglia dynamics in the developing brain and in response to injury, in an effort to formulate a hypothesis on how the intersection of these processes may modify long-term trajectories.

## Background

Traumatic brain injuries (TBI) can have devastating consequences on brain and mental health. Between Canada, the USA, and the European Union (EU), there are an estimated 46 million new TBI cases each year [1]. Among these, the vast majority (~ 80%) are classified as mild (mTBI) [2]. The highest age-specific rates for mTBI occurs in adolescents due to increased risk-taking behaviors and participation in contact sports [3], with sports-related mTBIs accounting for ~ 60% of all adolescent cases [4]. Importantly, individuals who sustain a single mTBI are at high risk for acquiring repetitive mTBIs (RmTBI) [5]. While preventative measures to reduce injury rates are improving, the incidence of adolescent RmTBI continues to grow with no current therapies to improve outcomes [6].

TBI pathophysiology encompasses primary and secondary injury cascades that collectively drive acute and chronic neurological damage and dysfunction. Defining the mechanisms of primary injury has been a major research focus, but the potential therapeutic window for intervention at this stage is limited, specifically in the case of mTBIs. This is because primary injury cascades initiate immediately following head trauma and unlike moderate-to-severe TBIs, diagnosis of mTBIs is often delayed. Therefore, concentrating research into the mechanisms controlling secondary injury cascades is an important step for developing strategies that aid recovery and improve the long-term neurological health in afflicted populations. A central component of secondary injury is neuroinflammation, a common process in a multitude of neuropathologies and neurodegenerative diseases [7–9]. Microglia, the brain’s resident immune cell population, play important roles in both brain development and neuroinflammation that accompanies TBIs. Since adolescence is a critical neurodevelopmental period, neuroinflammation, and specifically microglial activation following injury, may have detrimental consequences on long-term quality-of-life and even invoke early onset of neuropsychological disorders [10–12]. However, to date, the majority of TBI research has focused on adulthood, limiting our understanding of injury mechanisms during adolescence, and specifically those mediated by microglia in post-injury neuroinflammation. This review highlights major concepts surrounding neuroinflammation in TBI, the current understanding of adolescent brain development, microglial function in the developing nervous system, and the integration of these disciplines. By examining differences in microglia activities between adolescence and adulthood, we introduce novel concepts related to how these cells may differentially impact secondary injury following TBIs and specific functions unique to the adolescent state.

## Traumatic brain injury

TBIs are defined as insults to the brain caused by external forces resulting in acute or chronic neurological impairments. These injuries occur along a spectrum of severities (mild, moderate, and severe), pathologies, and clinical outcomes. Mild TBIs are among the most common in society (~ 80% of all diagnosed cases) and are frequently caused by sports-related collisions, falls, motor vehicle accidents, and war zone blast injuries impacting military personnel [13, 14]. They are typically characterized by a mechanical force delivered to the head, neck, or body that results in “coup” and “contrecoup” movements of the brain within the inside of the skull [15]. Len and Neary (2011) defined mTBI as a blow to the head and/or neck that causes rotational acceleration/deceleration of the brain, the onset of short-lived neurological deficits, potential loss of consciousness, and no skull fractures or macroscopic structural abnormalities assessed by neuroimaging. Mild TBIs are often classified as “concussions” in the clinical setting; however, not all mTBIs cause prototypical concussion [16]. Common neurological disturbances accompanying mTBIs range from acute symptoms including headache, nausea, dizziness, light/noise sensitivity, attention, concentration, and memory, to more long-term symptoms including irritability, sleep disturbances, anxiety, and depression [17–19]. When accounting for all acquired brain injuries, it appears that males experience more mTBIs than females with apparent sexually dimorphic symptomologies [20]. For example, when comparing male and female concussed soccer players, Covassin and colleagues found that females performed worse in visual memory tasks, had more total concussive symptoms (verbal and visual memory), and higher rates of migraine-induced cognitive fatigue and sleep disturbances up to 8 days post-injury [21]. Although most patients recover from post-injury symptoms within 7–10 days, a significant portion experience persistent symptoms that last for months, years, and in some cases for the remainder of their lives. These lingering symptoms have been termed post-concussive syndrome [22]. Moreover, individuals who acquire a single mTBI are at high risk for sustaining RmTBIs (due primarily to the nature in which the injuries are acquired) which can compound symptom severity and persistence [23]. RmTBIs are particularly relevant to individuals in sport and military environments where recurrent head collisions and blast injuries from explosions are frequently experienced. These individuals often return to play or duty (in the case of military personnel) before the brain has fully recovered, which can result in additive negative effects on long-term brain and mental health, and propel chronic neuropsychiatric disorders and neurodegenerative diseases [24, 25], such as mild cognitive impairment, Alzheimer’s disease (AD), and related dementias, and Parkinson’s disease (PD) [26, 27].

Adolescents have emerged as a prominent demographic for sustaining RmTBIs [28]. Remarkably, it is estimated that 1-in-6 youth experience a second injury within 2 years of their first [29]. Although males present more frequently with TBIs when accounting for all severities, among sex-comparable sports, adolescent females experience higher mTBI rates than males and often have worse symptomologies [6, 30], potentially due to differences in self-report [31]. To date, the majority of preclinical mTBI research has focused explicitly on adult injuries and neglected the female population [32]. While these studies have shed important light on pathophysiological mechanisms that dictate primary and secondary injury severity and the development of chronic neurological disorders, the specific, sexually dimorphic effects of RmTBI on adolescents, and the resulting impact on their acute and chronic neurological health remains largely unknown. Since adolescence is a critical neurodevelopmental period characterized by ongoing neuronal development, maturation, and fine-tuned circuit integration [33], RmTBIs sustained during this period may have severe consequences for life-long brain and mental health.

Immediately following a mTBI, the brain is thought to reside in a period of vulnerability where acquiring additional injuries can exacerbate neuropathology and accompanying neurological deficits. We currently do not know how long this window of enhanced vulnerability extends in humans, but preclinical RmTBI studies in rodents have demonstrated cumulative behavioral deficits and exacerbated neuropathophysiological hallmarks when administering consecutive injuries at varying time intervals. For example, Longhi and colleagues determined a critical time window for exacerbated behavioral deficits and neuronal dysfunction/damage when consecutive injuries occurred at 3–5-day intervals in adolescent male mice [34]. Subsequent studies have expanded this finding by altering RmTBI paradigms from multiple mTBIs per day for multiple days [35], to single mTBIs delivered every 24 h [36], 48 h [37], or 72 h [38]. Wright and colleagues directly compared the effects of a single mTBI or RmTBI in adolescent rats and noted cumulative effects of RmTBI on behavioral deficits, gene expression differences, and axonal integrity as compared to sham injuries with noted sex-differences [39]. Specifically, male rats exposed to RmTBIs had significantly worse deficits in short-term working memory whereas females displayed increased depressive-like symptoms. At the structural level, RmTBIs caused atrophy to the prefrontal cortex specifically in females whereas males had increased white matter tract damage in the corpus callosum [39]. To our knowledge, only one study has directly compared recovery from RmTBI in adolescent (post-natal day (P) 35) and adult mice (P120). While adult mice had reduced white matter volume compared to shams, no white matter changes were seen in adolescents following RmTBI. Despite the lack of gross anatomical changes, adolescent mice had prolonged behavioral deficits in motor and memory tasks up to 3 months post-injury [40]. This may suggest that secondary injury cascades accompanying TBI persist for days and even months following the initial injury, or that acute neuroinflammation causes chronic neurological changes, emphasizing the need to further characterize the temporal nature of cerebral vulnerability windows. In addition to these time-dependent effects, many environmental factors have been shown to influence the behavioral and molecular outcomes associated with RmTBI including sleep deprivation [41], diet (ketogenic diet: [42]; caffeine consumption [43]; monosodium glutamate (MSG) consumption [44]; alcohol consumption [45]), environmental enrichment [46], and exercise and anabolic steroid use [47]. Given the lack of knowledge surrounding cerebral vulnerability windows and the influence of various environmental factors on RmTBI pathophysiology during adolescence there is a significant need for future research in this area.

## TBI pathophysiology

### Diffuse axonal injury

Immediately following mTBI, primary injury cascades initiate that drive acute neurological deficits. Primary injury is caused by rotational acceleration and deceleration forces that cause differential velocities of white and grey matter (due to differing densities). The differential movement of white and grey matter imparts tensile and compressive strain to white matter tracts, which can sometimes result in shearing or tearing of axons. This is commonly referred to as diffuse axonal injury (DAI) (for reviews on DAI see [48, 49]). DAI primarily occurs throughout white matter tracts within deep and subcortical brain regions like the corpus callosum [50]. This may reflect vulnerabilities of white matter tracts that are organized in parallel oriented bundles, making them more susceptible to the tensile strain associated with rotational acceleration and deceleration of the brain [49]. DAI is a major hallmark of all severities of TBI [50–52]. The brain is normally resilient to stretching and recovers to normal shape; however, during repetitive injuries the brain can lose these elastic capabilities exacerbating DAI pathology [53].

**Fig. 1:**
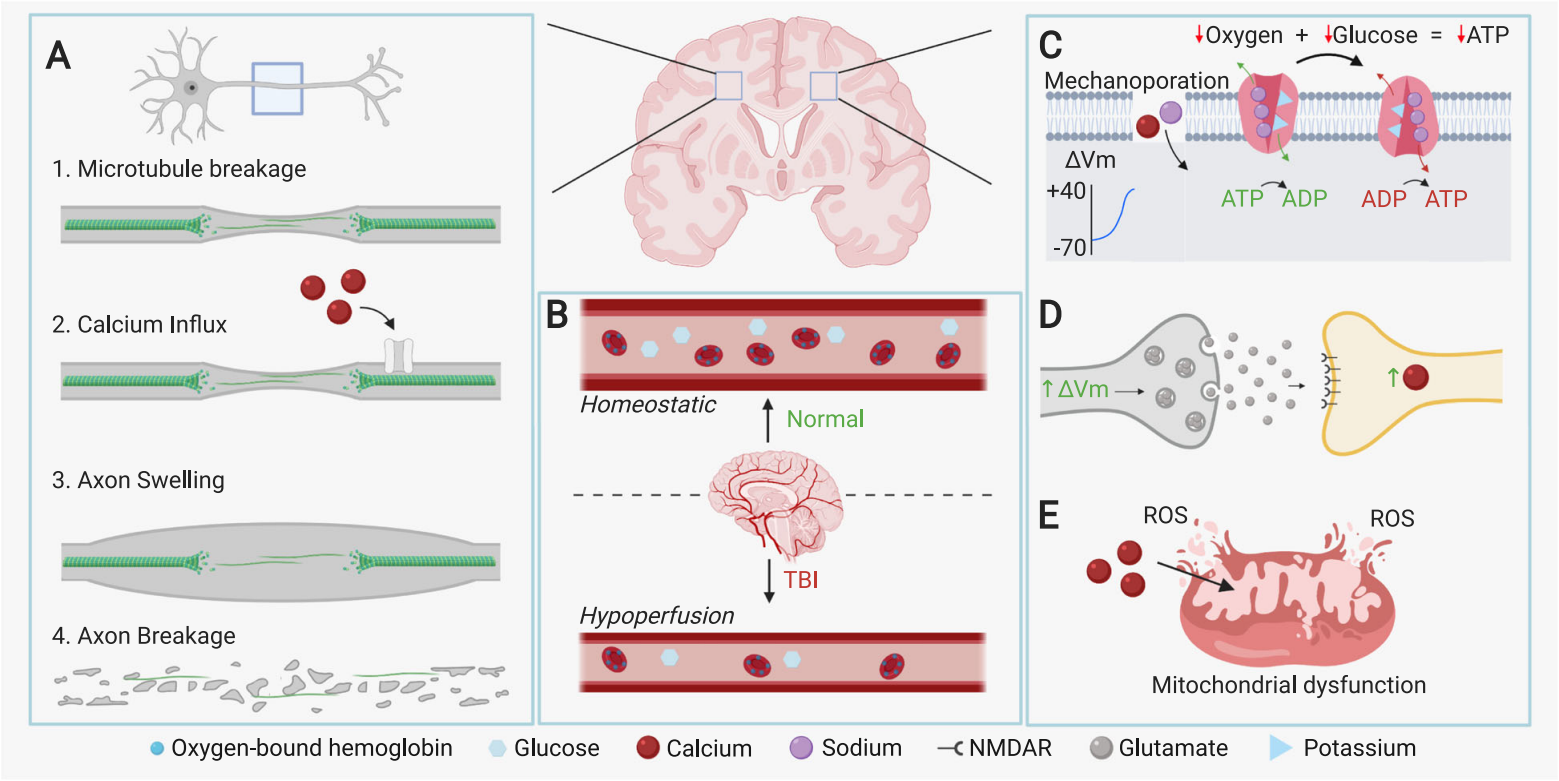
Primary and secondary injury cascades following TBI. **a** Diffuse axonal injury that results from the differential velocities of white and grey matter during a traumatic impact induces tensile strain and microtubule damage within axons. This event induces accumulation of microtubule transport proteins/cargo and calcium influx, resulting in axonal swelling and ultimately axonal degeneration. **b** TBI reduces cerebral blood flow through impaired autoregulation and increased vasoconstriction, thereby reducing glucose and oxygen throughout the brain. **c** Axonal stretching induces mechanoporation, facilitating depolarization via the influx of sodium and calcium ions. Additionally, reductions in oxygen and glucose delivery reduce neuronal ATP levels causing failure or reversal of ATP-dependent ion transporters/pumps such as the sodium/potassium ATP pump. This further exacerbates ionic dysregulation by exporting potassium and importing sodium ions. **d** Ionic imbalances which lead to depolarization of pre-synaptic neurons result in dysregulated glutamate release into the synaptic cleft, which over-activates NMDAR receptors and increases calcium influx into post-synaptic neurons (termed excitotoxicity). **e** Unregulated calcium influx drives neuronal death through mitochondrial dysfunction and the release of reactive oxygen species (ROS)

There are a number of pathophysiological hallmarks related to DAI (Fig. 1a). In the axon, shearing and tearing can drive aberrant influx of extracellular calcium causing neurofilament compaction, microtubule disassembly, interruption of axonal transport, the accumulation of transported organelles, and finally axonal swelling and axotomy [54–56]. While not characteristic of all time frames, axonal swelling is typically seen in the acute post-injury phase where axons can experience a 30-fold increase in size from normal physiological conditions [56] leading to altered conduction velocities [57, 58]. Axons within a tract can also become misaligned due to cytoskeletal failure as a result of broken microtubules [56]. For example, in an in vitro model, stretching of axons caused microtubule breakage at the crests of axon undulations (smooth wave-like crests and troughs) [56]. At more chronic time points, axons are often shrunken, display axonal varicosities (swellings along the length of an axon), and formation of terminal axonal bulbs (singular axonal swelling resulting in complete axonal disconnection), common to Wallerian degeneration [56, 59, 60]. Collectively, these pathological changes to white matter tracts interrupt normal neurotransmission and negatively impact neural circuit dynamics. Moreover, axonal injury can spread over time further compromising healthy brain function. While the precise mechanisms driving the spread of injury are not clear, this phenomenon may be explained in part by the actions of secondary injury cascades.

### Secondary injury cascades

The accelerative and decelerative forces experienced by cell membranes during TBI, combined with primary DAI pathology, can initiate secondary injury cascades that have additional negative effects on neuronal health and function (for in-depth reviews of secondary cascades, see [61, 62]. Secondary injury cascades can initiate within minutes of injury and extend for months. They are characterized by dysregulated cerebral blood flow (CBF) [63], altered metabolic and cellular homeostasis, ionic dysregulation [64], mitochondrial dysfunction [65], neuronal atrophy, cell death, and neuroinflammation [66] (Fig. 1b). Detailing the entirety of secondary injury cascades following mTBI would be a tall task and reviews of this topic have been identified above. As it is important to have a broad understanding of the neural environment following injury, this review will touch on a few of the most important components of secondary injury cascades, before delving into an in-depth review of neuroinflammation (specifically microglial response) in Section 3.3.

In humans, mTBIs generally cause acute periods of reduced CBF up to 1 day post- injury [67], although CBF regulation is complex and may vary by age and injury severity. Cerebral hypoperfusion is primarily driven by impairments in cerebrovascular autoregulation (vascular constriction/dilation in response to changes in perfusion pressure to maintain constant blood flow) resulting in significant reductions in oxygen and energy substrate (e.g., glucose) delivery to the brain (Fig. 1b) [16, 68]. Interestingly, human and rodent TBI studies have collectively reported acute hypermetabolism following brain injury followed by variable periods of hypometabolism depending on the severity of injury [69–72]. Prolonged reductions in oxygen and glucose transport to the brain, along with hypometabolism, depletes ATP stores that are vital for maintaining charge separation across neuronal membranes and efficient neurotransmission (Fig. 1c). In experimental rodent models of severe fluid percussion TBI, global reductions in CBF were observed as early as 15 min post-injury and recovered within 2-h [73]. In both human and rodent RmTBIs, alterations to CBF and metabolism are evident and vary depending on the mechanism and number of injuries [74–76]. For example, following murine RmTBI, decreased cortical CBF was observed accompanying microglia activation, in part due to the upregulation of cytokines such as RANTES, interleukin (IL)-13, IL-10, and IL-15 [77]. Combined with reductions in CBF, axonal stretching can induce mechanoporation and activate mechanosensitive ion channels, further exacerbating ionic imbalance across neuronal membranes perpetuating depolarization (Fig. 1c) [78]. Mechanoporation typically occurs in the acute phase following TBI and is characterized by the formation of small non-selective pores in the lipid membrane that allow ions to travel down their electrochemical gradients [78]. Collectively, the ionic imbalance in affected neurons can alter the surrounding neural environment causing secondary ionic disturbances in nearby uninjured neurons [79]. This membrane dysfunction can persist for several hours post-injury [79]. In response to reduced CBF and mechanoporation-dependent depolarization of neurons, aberrant neurotransmitter release ensues that can lead to excitotoxicity and neuronal death. In general, this is thought to be driven largely by overactivation of post-synaptic NMDA receptors in response to excessive glutamate release, pathological influx of Ca^2+^ ions, and subsequent mitochondrial dysfunction (Fig. 1c). Although our understanding of the molecular mechanisms driving these injury cascades has broadened, the precise timing of CBF alterations, cytotoxic ionic imbalances, and accompanying pathways driving neuronal death/dysfunction have not been fully elucidated and appear to vary depending on the type and extent of injury. Notwithstanding, it is now clear that the immune system plays central roles in the regulation of primary and secondary injury cascades. Specific modulation of the post-traumatic neuroinflammatory response may therefore provide unique opportunities to limit the extent of neural dysregulation/damage accompanying TBI and improve recovery.

### Neuroinflammation

Pathophysiological signaling processes that accompany TBIs can initiate complex, dynamic neuroinflammatory responses that modulate both recovery and secondary injury cascades. Neuroinflammation is mediated by a number of different cell types including local glial cells (microglia, astrocytes), cerebrovascular endothelial cells, and peripheral immune cells (macrophages, neutrophils, T cells, B cells, etc.) [80].

Microglia are thought to be the first responders to injury. They detect alterations in their local environments by activating and synthesizing/secreting a plethora of signaling molecules that coordinate diverse effector functions such as phagocytosis of cellular debris and cytotoxic molecules, modulation of neuronal protein expression and activity, and orchestration of neural repair pathways [81]. Microglia are highly plastic cells, able to rapidly change along a spectrum of pro-inflammatory (M1-Like) and anti-inflammatory (M2-like) phenotypes on an “as need” basis [82–84]. Under normal homeostatic conditions, microglia primarily exist in a surveillance state, sampling the extracellular milieu on a constant basis through their motile, ramified processes. In response to tissue damage or infection, these cells are activated, retracting their processes and adopting amoeboid morphologies reminiscent of peripheral macrophages [85]. Activated microglia that adopt classical M1-like phenotypes primarily synthesize and release pro-inflammatory molecules including IL-1β, IL-6, IL-12, and tumor necrosis factor α (TNFα) cytokines and CCL2 and CXCLR9 chemokines [86]. These pro-inflammatory cytokines induce alterations in intracellular Ca^2+^ dynamics that can modify synaptic plasticity, neurotransmitter release, and neuronal excitability [87, 88]. Conversely, M2-like microglia are considered “alternative” activated states promoting anti-inflammatory profiles (induced by IL-4, IL-10, IL-13, and transforming growth factor-β) involved in resolution of neuroinflammation through increased phagocytic activity and tissue repair [89]. It is now clear that microglia are highly heterogeneous and can adopt a variety of phenotypes depending on changes to their immediate neural environments. As such, it is not surprising that these cells play dynamic roles in neuroinflammation, influencing both injury resolution/tissue repair and further dysfunction/destruction.

**Fig. 2:**
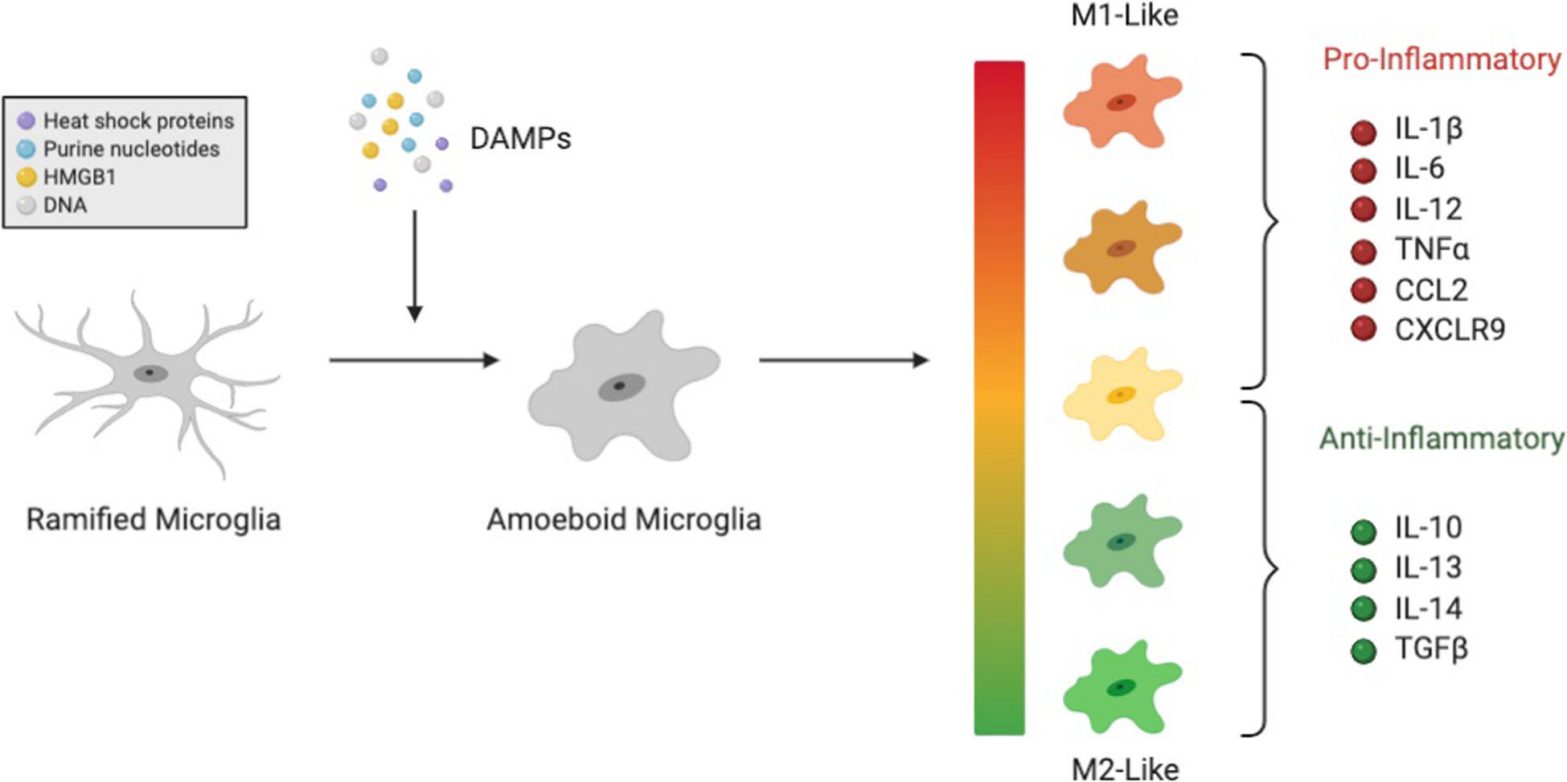
Microglia phenotypes following TBI. DAMPs released by injured, damaged, and/or degrading brain cells activate microglia from homeostatic surveillance/ramified phenotypes to activated inflammatory/amoeboid phenotypes. Activated microglia may differentiate along a spectrum of pro-inflammatory (M1-like) and anti-inflammatory (M2-like) phenotypes, synthesizing and releasing a plethora of pro- and anti-inflammatory cytokines/chemokines, respectively

Within the acute post-injury window, disruptions in neuronal membranes by mechanical forces and DAI cause release of damage-associated molecular patterns (DAMPs; e.g., ATP, HMGB1 and heat shock proteins) that can signal through receptor-dependent mechanisms to activate microglia, and alter inflammatory phenotypes and effector functions (Fig. 2) [90, 91]. In addition to these immediate responses, microglial-derived signaling molecules and DAMPs can modulate blood-brain barrier (BBB) function in the cerebral vasculature [90]. While the central nervous system (CNS) has historically been considered an immune-privileged organ due to this barrier and absence of classical antigen presenting cells, it is now well recognized that peripherally restricted immune cells can gain access to the brain parenchyma in cases of injury and/or infection [92, 93]. Like many other neuroimmunological diseases, TBIs can disrupt the BBB and permit the invasion of peripheral immune cells that play dynamic roles in the post-injury inflammatory profile (for review on mechanisms of BBB function/permeability in TBI see [94]).

In experimental models of TBI, timing of BBB disruption often occurs in a biphasic manner and is dependent upon injury severity and the method of injury induction [95]. In models of moderate-to-severe TBI, BBB permeability has been observed immediately following injury (~ 2 h) and persists for up to 7 days post-injury [63, 95]. Conversely, in mTBI and RmTBI, there is high variability in observable BBB disruption, with some studies reporting robust disruption and others reporting no observable changes [60, 63, 96–99]. Furthermore, diffuse injuries may result in sexually dimorphic responses whereby males are more prone to BBB disruption than females [97, 100].

Congruent with the dynamic nature of BBB permeability, the infiltration of peripheral immune cells appears to occur in a cell type-specific and time-dependent manner. Circulating neutrophils are among the first cells to infiltrate, usually within the first hour and peaking by 24 h post-injury, initiating enhanced inflammatory signals [101, 102]. Monocyte-derived macrophages enter the CNS within 24 h of injury and peak at approximately 96 hours, readily crossing the BBB aided by neutrophils [103–105]. T cells and dendritic cells, peaking at ~ 72 h, have similar profiles to monocytes but in lower numbers [106, 107]. Together, the spatiotemporal dynamics of microglia, precise timing of peripheral immune cell infiltration, the identity and phenotypic polarization of these cells, and the coordinated signaling mechanisms they impart shape the neuroinflammatory environment and dictate dichotomous aspects of neural repair and secondary injury.

While we appreciate that the inflammatory response in TBI is complex and multifaceted, this review focuses on the differential roles for microglia in adolescent mTBIs. For in depth review of global neuroinflammatory dynamics in TBI see Simon et al., 2017 [108]. We believe that there are two key areas of research missing within the literature regarding the microglial inflammatory response following TBI. First, there is a lack of representation of the adolescent time period with most studies to date focusing on adulthood, and second, there is a gap in knowledge with respect to females, where the majority of studies have focused solely on males. Given known sex differences in microglial development and injury responses [109–111] along with age-specific effector functions, we believe that these cells play important, differential roles in neuroinflammation accompanying adolescent RmTBI.

## Adolescence

Adolescence represents a critical developmental period between childhood and adulthood, generally categorized from 8 to 20 years of age in humans [112], 30 to 60 months in non-human primates [113], and 35 to 60 days in rodents [114]. This dynamic developmental period encompasses a multitude of changes including refinement of higher-order cognition [113], brain maturation [33], the onset of puberty [115], changes in social and risk-taking behaviors [12], and onset of major disorders such as schizophrenia [10], substance-abuse [11], and mood disorders [116]. The start of adolescence is generally characterized by the onset of puberty (although adolescence and puberty are exclusive terms) [113]. Interestingly, the hormonal events associated with puberty influence brain maturation in females before males [117]. While sex hormones are responsible for sexually differentiating neural circuits during embryogenesis, during puberty, they activate reproductive behaviors and organize neural circuits [115].

### Adolescent brain development.

Adolescent maturation is a complex process that culminates in novel and complex social and environmental interactions and experiences. Specifically, it represents a period of social autonomy, sensation-driven behavior, and sexual maturation [118]. Throughout adolescence the brain is extremely plastic and undergoes robust experience-dependent rewiring influenced by coordinated dendritic spine formation and elimination, and myelinogenesis [119, 120], (for review on adolescent brain development, see [121]). By adolescence, total brain volume is relatively stable and remains this way into adulthood. However, this does not explicitly imply that all brain regions undergo linear development. Grey matter (unmyelinated axons, dendrites) decreases during adolescence in a region-specific manner [121, 122]. These grey matter changes appear to be dependent on significant region and sex-specific changes in synaptic pruning [123, 124]. Interestingly, testosterone has been positively correlated with grey matter volume whereas estradiol levels have been negatively associated [125]. In contrast to grey matter, white matter volume expands throughout adolescence in a sex-specific manner, whereby females demonstrate earlier developmental changes, but males appear to show steeper age-related increases [126, 127]. Given that adolescence is a critical period of learning, increased social autonomy, and complex social interactions [128], it is likely that white matter tract development is continually altered throughout this period. In both longitudinal and cross-sectional studies, fractional anisotropy (a measure of white matter integrity) exhibits both age and region-specific changes [129]. In one particular study, region-specific increases in fiber integrity were noted in all fiber tracts throughout the brain measured (arcuate, cingulum, fornix, genu, inferior fronto-occipital fasciculus, inferior longitudinal fasciculus, splenium, and uncinate) when examining adolescents between 9 and 12 years of age [129]. Importantly, microglia play important dynamic roles in the developing CNS and their dysregulation may have profound effects on the adolescent neurological state.

## Microglia and the developing CNS

Microglia are among the most abundant and versatile glial cells in the CNS, encompassing ~ 10–15% of total glial cells [130]. While it has classically been thought that microglia primarily functioned in CNS immune responses and homeostasis, it is now known that they play central roles in shaping CNS development by promoting neuronal survival, inducing programmed neuronal death, and fine tuning synaptic connections through synaptogenesis and dendritic spine pruning [131, 132]. Dysregulation of microglial function has been implicated in a number of neurological disorders and pathologies including obsessive compulsive disorder [133], AD [134], PD [135], chronic traumatic encephalopathy (CTE) [136], and brain infections [137]. Microglial dysfunction has also been implicated in aging [138]. Aging-related dysregulation may impair microglia’s ability to regulate homeostasis by altering the balance between surveillance and activated phenotypes along the M1-like/M2-like spectrum [138, 139]. Given microglia play central roles in CNS development in utero and immediately following birth, it stands to reason they would also shape the CNS during other critical periods of neurodevelopment, including adolescence. This is especially relevant when considering the substantial white matter tract development in the prefrontal cortex (PFC) that occurs in this age group [122] [126]. Uncovering age-related differences in TBI pathophysiology and specific functions of microglia could therefore increase our understanding of the differential impact of sustaining these injuries during adolescence or adulthood.

### Microglia development

Microglia are the first glial cells to colonize the CNS, however, unlike the neuroectodermal origin of most neural cells, microglia are derived from the yolk sac (for reviews, see [140]). The most conclusive evidence for this was determined by Ginhoux and colleagues (2010). During early embryogenesis, they populate the brain in an amoeboid morphology, which facilitates migration, and share many surface antigens and effector functions with peripheral blood-borne immune cell populations [141]. In rodents, amoeboid microglia transition towards a ramified morphology around embryonic day (E) 10.5 and complete ramification by P28 [142]. During this period, microglia are also proliferating. Microglial proliferation is a continual process beginning in the early postnatal days of murine life and reaching maximal concentration around two weeks of age [143]. By 18 weeks of age in humans, most microglia display ramified morphologies and have dispersed throughout the CNS [144]. These mature microglia display smaller cell bodies and longer, more ramified processes compared to immature microglia [145]. The transition from amoeboid to ramified morphologies appears to be dependent on the presence of other CNS resident cells, notably neurons and astrocytes [146–150].

Notwithstanding, there appear to be spatial differences in microglia morphology. For example, in subcortical white matter microglia display more amoeboid morphologies, whereas cortical microglia display diffuse distribution and ramified morphologies [131]. During development, microglia in murine models form hotspots and cluster in white-matter tracts with distinct spatiotemporal patterns of distribution and expression of different cellular markers [81] [141, 144]. The clustering on white-matter tracts suggests microglia play an important role in axonal growth, guidance, and myelination [141]. Indeed, through regulation by cytokines and chemokines (i.e. IL-34, CXCL12, and CX3CL1), microglia promote axonal outgrowth in regions such as the corpus callosum [151, 152]. Throughout adulthood, microglia exhibit longevity independent of replenishment by bone marrow-derived progenitors, owing to their ability to undergo self-renewal [153]. Transplants from bone marrow derived myeloid cells reconstituted less than 10–20% of total microglial cells, indicating that once microglia enter the CNS they are self-renewing and do not re-infiltrate from the periphery [142]. Self-replication means microglial density remains fairly consistent throughout life, although there are region-specific and pathological turnover rates [154].

Recently, sex differences in microglial morphology, maturation, and function have come to light. In the early postnatal period, males exhibit region-specific (amygdala, hippocampus, nucleus accumbens, preoptic area) increases in microglia number, while at the beginning of adolescence (P30), females have more microglia in those brain regions [110, 155]. Production of testosterone by testes in the late gestation period may drive the early microglial increases in males since ovaries are largely silent at this time [155]. Once colonization is established, microglia contribute directly to many facets of brain development and sexual differentiation.

### Microglial effector functions in the developing CNS

Microglia are key contributors to normal CNS development and function, providing important multifaceted effector functions that shape the CNS throughout life. The precise timing of microglia emergence in the CNS corresponds with the birth of neurons [131, 152]. Accordingly, in the prenatal and perinatal brain, microglia regulate neurogenesis and maturation of developing neurons by controlling the balance between neuronal death and survival, phagocytosing neuronal progenitors, coordinating axonal outgrowth, and promoting neuronal fasciculation [156–158]. During embryogenesis, microglia regulate neurogenesis at the terminal stage of cortical development by phagocytosing progenitor cells in the ventricular and subventricular zones [152]. Support for this function was determined by pharmacologically depleting microglia, which resulted in an increase in progenitor cell number. Conversely, activation of microglia from their ramified, homeostatic state by maternal immune activation (injection of lipopolysaccharide) caused a decrease in neural progenitors [152]. This neurogenic role does not conclude in utero, but continues throughout the post-natal period regulating neural development in the subventricular zone of the developing cerebral cortex [152]. During development microglia also play dual roles that control differentiated neuronal survival and elimination. Over half of the neurons present at birth are eliminated. Microglia directly regulate survival by releasing trophic factors (i.e., insulin-like growth factor-1 promoting the survival of layer V neurons) [131] and eliminate specific neurons undergoing programmed cell death via coordinated microglia-dependent phagocytosis [159].

In the post-natal brain microglia modulate synapse number, maturation, and survival, all of which collectively influence neuronal activity [160]. Most notably, recent discoveries have highlighted microglia as direct regulators of synaptic pruning [132, 160, 161]. Throughout normal development, neurons create excess synaptic connections, and it is the role of ramified microglia to prune unnecessary connections and strengthen remaining synapses [160]. Removal of excess synapses is vitally important for the development of proper neuronal connections and the integration of higher-order circuits that control healthy CNS function [162–164]. To date, microglial-dependent synaptic pruning has been observed in numerous brain regions such as thalamus, cerebellum, olfactory bulb, and hippocampus (HPC) [165]. The functioning of microglia in synaptic pruning during development is highly responsive to experience [166, 167]. Evidence for microglial experience-dependent synaptic pruning comes from experiments in the developing visual cortex [132]. In short, Tremblay and colleagues found that under normal development microglia clustered around growing dendritic spines and were in direct contact with synaptic clefts. In response to altered sensory experience (light deprivation) and re-exposure, microglia were less motile, increased phagocytosis of synaptic elements, and localized to larger dendritic spines [132]. This experience-dependent synaptic pruning implicates microglia in synaptic remodeling.

**Fig. 3:**
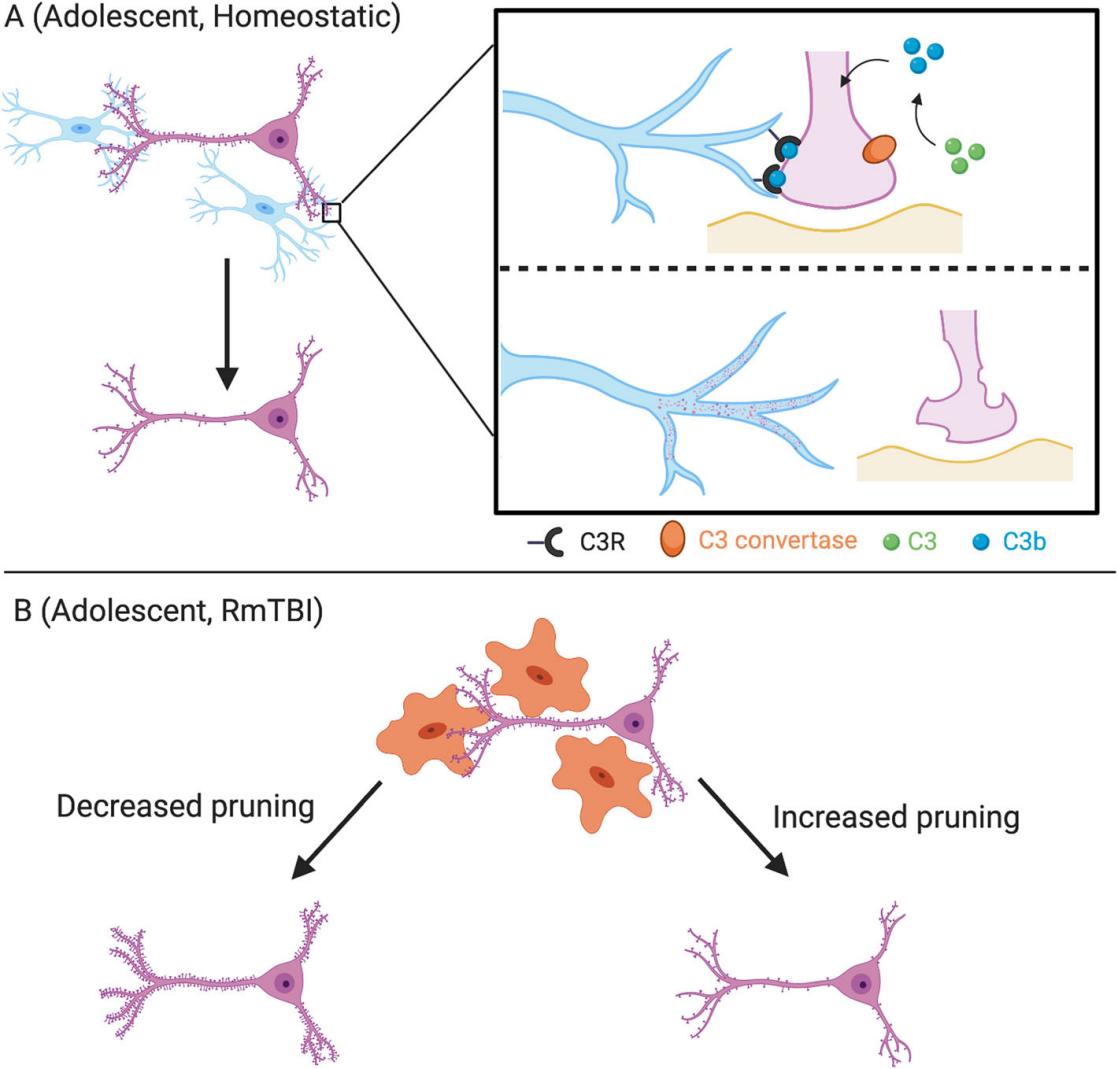
Complement-mediated synaptic pruning by microglia in the homeostatic and RmTBI adolescent brain. **a** Under homeostatic conditions, the complement protein C3 is converted to C3b by C3 convertase which tags unnecessary or weak synapses for pruning. Microglia, which highly express the C3 receptor (C3R), bind to synaptically tagged C3 molecules facilitating microglia-mediated pruning of pre-synaptic terminals. **b** Following RmTBI, microglia-mediated synaptic pruning may be either increased or decreased compared to homeostatic conditions. Ramified microglia are thought to be more efficient at synaptic pruning compared to amoeboid microglia, which could reduce pruning during adolescence. Alternatively, increased expression of C3 following RmTBI may facilitate increased synaptic pruning by activated/amoeboid microglia. Collectively, alterations in synaptic pruning caused by RmTBIs may directly influence synaptic density and overarching neural development and health

But how do microglia determine which synapses to prune? Recently, the complement system has been implicated in regulation of microglial-dependent synaptic pruning (Fig. 3). The complement system comprises a series of proteins, C1-to-C9, that are part of the innate immune system with roles in cytokine production, vascular permeability, recruitment of macrophages, and opsonization (for review, see [168]). The specific complement proteins C1q, C3, and C4 have been directly linked to synapse engulfment [169]. Microglia express receptors for C1q (C1qR) and C3 (C3R or CD11b) [170] that coordinate the mechanisms for selective engulfment of pre- or post-synaptic terminals. First, microglia recognize C1q tagged on post-synaptic dendrites (through their C1qR) to initiate phagocytosis of this structure. Second, neuronal upregulation of C1q cleaves C3 into C3b which is displayed on pre-synaptic elements for targeted phagocytosis [170]. Knocking out C1q results in deficits in synaptic pruning causing excessive innervation of lateral geniculate nucleus neurons [169]. Tagging of pre- and post-synaptic elements by specific complement proteins is thought to be regulated by activity. It has been proposed that less active (weaker) synapses are selectively tagged with complement components to signal their engulfment and strengthen remaining connections. Alternatively, it is possible that all synapses express complement components but some also harbor complement regulatory proteins that prevent recognition by microglia [171, 172]. Nonetheless, it is clear that synaptic pruning through the complement system is tightly regulated and likely restricted to key developmental stages [109, 169]. However, Hong and associates determined that in rodent models of AD, C1q, and C3 are expressed on synapses before β-amyloid plaque formation and associated synaptic loss, while blockade of the complement cascade in this system protected against synaptic loss [173]. This observation may suggest that complement-mediated synaptic pruning by microglia drives the early synaptic and cognitive impairments that often precede overt plaque formation in AD. To date, the majority of studies on complement-mediated synaptic pruning by microglia have focused on early developmental periods, but an important recent study extended these observations into the adolescent stage. Kopec and colleagues determined that C3 and microglial C3R mediated synaptic pruning in dopaminergic neurons in the nucleus accumbens of adolescent male rats [174]. Interestingly, synaptic pruning in females was not mediated by C3 and microglial C3R. When C3-C3R interactions were pharmacologically blocked, microglia exhibited decreased phagocytic activity resulting in increased rodent social play behavior in a sexually dimorphic manner.

## Age- and sex-dependent microglia dynamics in TBI pathophysiology

### The dual role of microglia in neuroinflammation

Microglia-dependent inflammatory processes in TBI are evident in both focal and diffuse injuries (for review, see [108]). As a result of neuronal membrane damage/disruption, released DAMPs activate microglia, causing proliferation and migration to sites of injury in an effort to “wall off” damaged neurons, phagocytose cellular debris, and clear cytotoxic molecules from the extracellular milieu [175, 187]. The heterogeneous effects of mTBI and RmTBI on microglial reactivity are summarized in Table 1. Nonetheless, it is generally accepted that while the acute microglial response has neuroprotective effects [108], when these cells reside in chronically activated states, they contribute to the window of cerebral vulnerability and compound the negative effects of repetitive injuries on neurological health.

**Table 1:** Microglia activation following TBI

Reference	Strain/sex/age	Model (severity)	# of impacts/ICI	Microglia marker	Time PI	Regions	Findings
[37]	C57/male/9-15 months	Controlled impact	5/48 h	Iba1	6 months, 12 months	Cortex (retrosplenial, sensormotor, motor), CC, CA1, DG	Cortex: no difference at 6 or 12 months; CC: Increase at 6 months and 12 months; CA1and DG: no change
			1	Iba1	6 months, 12 months	Cortex (retrosplenial, sensorimotor, motor), CC, CA1, DG	Cortex: no difference; CC: Increase at 12 months; CA1 and DG: no difference
[38]	Sprague-Dawley/male and female/P30	Lateral impact	3/3 days	Iba1	16 days	VMH	Increase following RmTBI in males only
[40]	C57/male/P35	Mod. weight drop	7/9 days	Iba1	3 months	CA1	Increase in both ages with no difference in adolescent’s vs adults
	C57/male/P120	Mod. weight drop	7/9 days	Iba1	3 months	CA1	
[98]	C57	Mahmood weight drop	5/24 h	Isolectin B4	30 days	HPC	No activation
			10/24 h	Isolectin B4	30 days	HPC	No activation
[175]	Long-Evans/male/adult	mLFP	1	CD68	4 days	Ips and Con perirhinal cortex, parietal cortex, temporal cortex	Increased expression in all regions
					32 days	Ips and Con perirhinal cortex, parietal cortex, temporal cortex	No differences
[176]	C57/male/P120–150	Mod. CCI	30/ 5 on 2 off	Iba1, CD68	1 day, 60 days, 365 days	Optic Tract and LGN	Elevated at all time points
[177]	C57/male/P84	Mod. CCI	42/ 6 per day/2h	CD68	7 days, 1 month, 6 months	Cortex, Amy, DG, CA1, CA3	7 days: Increase in cortex, con amy, CA3; 1 month: increase ips cortex, ips amy, ips/con DG; 6 months: Increase ips/con cortex, ips/con amy, ips/con DG, ips/con CA1, ips/con CA3
[178]	C57/male/P60–90	Mod. CCI	2/24 h	Iba1	2 days, 4 days, 7 days, 14 days, 28 days, 49 days	Ips cortex, Ips DG, CC, Ips Thalamus	Ips cortex: Increase 4/7/14/28d; Ips thalamus: Increase 4/7/14/28 days; CC: Increase 4/7/14/28/49 days; Ips DG: Increase 2/4/7 days; Con CA: Increase 4 days
[179]	C57/ male/P70	Controlled impact	1	Iba1	24 h, 10 days	Cortex, CC, brainstem	Cortex: no difference; CC: moderate both times; CA1: present both times; brainstem: no difference
			5/48 h	Iba1	24 h		Cortex: moderate increase; CC: intense increase; brainstem: no difference
[180]	BALB/c/male/P90	Midline diffuse FPI (moderate)	1	Flow: CD14; Immuno: Iba1	Flow: 4 h, 72 h; Immuno: 30 days	Flow: Cortex, HPC; Iba1: DG, PCX, PFC;	Flow: CD14 microglia increases at 4 and 72; Iba1: 30 days increase in DG and PCX but not PFC
[181]	Wistar/female/P56	CCI (moderate-to-severe)	1	Flow: CD40, CD68, CD163; Immuno: CD68, C163, CD68, Iba1	1 day, 3 days, 5 days, 7 days, 14 days, 30 days	Cortex	Flow: CD163 increase 3 days and 5 days; CD40 and CD68 no difference Immuno: CD68: increase at 5/7/14 days; Cd163: Increase 5 and 7 days; CD68: No difference; Iba1: Increase 7 days
[182]	C57/male/P120–150	Closed-headed impact model of engineered rotational acceleration (CHIMERA)	1	Iba1	6 h, 1 day, 2 days, 7 days, 14 days	Optic tract, olfactory nerve, CC, brachium sup. Colliculus	Optic tract: increase 2/7/14 days; olfactory nerve: increase 1/2/7/14 days, CC: increase 2/7/14 days; Brachium: increase 2/7/14 days
[183]	Long-Evans/male/young adult	LFP	1,3,5/5 days	ED1	24 h	Injured cortex	Increase following 3 and 5 injuries
					8 weeks	Injured Cortex	Increase following 5 injuries
[184]	C57/male/P60	Mod. weight drop	7/9 days	Iba1, CD68	24 h, 7 days	Fimbria white matter and CA1	Increase in Iba1 and CD68 at 24 h and 7 days in both regions
[185]	C57/male/P60–90	Mod. weight drop	7/9 days	Iba1	3 months	Fimbria white matter and CA1	Increase in both regions
[186]	Sprague-Dawley/male/P18	Mod. CCI	1	Iba1	7 days	Cortex, external capsule, amygdala	No difference
			2/1 day	Iba1	7 days	Cortex, external capsule, amygdala	No difference

Abbreviations: *Amy* amygdala, *Con* contralateral, *CC* corpus callosum, *DG* dentate gyrus, *HPC* hippocampus, *ICI* inter-injury interval, *Ips* ipsilateral, *LGN* lateral geniculate nucleus, *LFP* lateral fluid percussion, *Mod* modified, *PCX* parietal cortex, *PFC* prefrontal cortex, *VMH* ventromedial hypothalamus

### Chronic neuroinflammation and microglial priming

Given the known homeostatic function of microglia in white matter tract development and synaptic organization, RmTBI-induced alterations to adolescent microglia may shift these cells to activated inflammatory states limiting their homeostatic role in normal development [81, 161]. Microglia can be chronically activated well after functional recovery from primary injuries, residing in a “primed” state that may exacerbate inflammatory responses with subsequent injuries or inflammatory stimuli [188, 189]. In the context of mTBI and the associated risk of sustaining RmTBIs, the priming of activated microglia is an important consideration for translation of preclinical studies to the clinical setting [175]. As mentioned previously, the precise recovery window following TBI appears to vary with age, injury severity, and other genetic and environmental factors. Intriguingly, microglial priming places these cells in sensitized states where successive mTBIs promote detrimental inflammatory functions. In support of this, experimental priming of microglia with lipopolysaccharide (LPS), which activates microglia through toll-like receptor (TLR) signaling, significantly increased the number of activated microglia for up to 28 days following RmTBI, compared to single injuries or sham controls [190]. Similarly, LPS priming of microglia 5 days following single mTBI compounded behavioral deficits (increasing depressive-like symptoms and impairing cognition) and exaggerated pro-inflammatory microglia phenotypes up to 3 months following injury [191]. Furthermore, microglia priming is exacerbated by increasing concentrations of IL-1β and TNFα, known endogenous signaling molecules elevated in the post-traumatic brain [180]. This priming effect may be best exemplified in the context of aging.

Microglia slowly transition from ramified surveillance states to activated phenotypes and increase in density throughout aging [192, 193]. Aging microglia also appear to lose the ability to transition between surveillance and activated states and tend to reside in more activated phenotypes as aging progresses [194]. In the preclinical setting, Kumar and colleagues utilized a controlled cortical impact (CCI) model of mTBI in adult (3 months) and aged (24 months) mice, identifying increases in M1-like and M2a-like microglial phenotypic markers and the presence of bushy/amoeboid microglia in the HPC, cortex, and thalamus of aged animals 24 h post-injury as compared to adult cohorts [195]. Prior work by Sandhir and colleagues reported that aged mice subjected to single CCI displayed increased Iba1 and CD11b mRNA and protein levels up to 3 days post-injury in the HPC, with a prolonged return to baseline as compared to adult mice [194]. Moreover, a complementary study utilizing a CCI model of RmTBI in aged mice revealed significant increases in Iba1 reactive microglia in the corpus callosum (but not HPC or cortex) 6 months post-injury which persisted for up to 12 months [37]. Consistent with these experimental studies, human patients with severe TBI may exhibit continued axonal degeneration and chronic microglia activation up to 18 years post-injury [196, 197]. Chronic microglial activation has also been reported in impact sport athletes following retirement suggesting that this phenomenon is not restricted to severe TBI, but also a hallmark of RmTBIs [198]. Collectively, both preclinical and clinical studies support the idea that microglia not only participate in acute inflammatory responses following brain injury, but can adopt primed, activated phenotypes that persist chronically and may play central roles in the pathophysiology accompanying cumulative TBIs. However, the characteristics of microglia activation in adolescent mTBI remain largely unknown and to our knowledge there have been no studies examining the impact of altered microglia dynamics during adolescent RmTBIs on adult outcomes.

### Microglia responses in adolescent TBI

Current literature regarding microglia-dependent inflammatory responses in adolescent RmTBI is sparse and requires further investigation. However, in a model of pediatric (P18) RmTBI, researchers identified more amoeboid microglia in the cortex, HPC, and amygdala with single or RmTBI on post-injury day 7. However, no differences were noted in total quantity of Iba1 positive microglia when compared to sham rats on post injury days 7, 21, or 92 [186]. These observations suggest that single and RmTBI caused microglia activation with no overt gliosis. Similar to the findings by Fidan, Wu and colleagues saw no differences in Iba1 expression in the cortex, corpus callosum, or hippocampus, 4 h and up to 1 year, between RmTBI and sham animals [199], which corroborates findings from adult research. It is important to note that while Iba1 is commonly used to identify microglia, it is also expressed by peripheral macrophages and may not be an ideal marker for microglia in injury models that cause BBB permeability and infiltration of peripheral cells. Juvenile (P21) mTBI induced region- and sex-dependent differences in microglial responses, whereby, males experienced higher activation of microglia compared to females, especially in regions affected by DAI [200]. Mannix and colleagues utilized a modified weight drop model of RmTBI to compare microglia-dependent outcomes in adolescent (P35) or adult (P120) wild-type C57Bl/6 mice. To our knowledge, this was the first closed-headed RmTBI model to directly compare adolescence and adult subjects. Adolescents exposed to RmTBI displayed similar magnitudes of gliosis to adults with RmTBI [40]. However, they did not monitor adolescent development following RmTBI into adulthood to determine long-term behavioral deficits or microglial reactivity. Given significant brain maturation is occurring during adolescence and the role microglia plays in synaptic pruning and overarching spine density, it is possible that RmTBI may alter normal neural development. While not directly analyzing microglial involvement, a number of studies have displayed increased spine density following adolescent RmTBI [43, 201–203]. Reductions in synaptic pruning may result from altered microglial functioning. Chronically activated microglia following RmTBI may be improperly regulating (or inhibiting) homeostatic functioning, leading to decreased synaptic pruning and increased spine density. It is known that alterations in synaptic density are hallmarks of a number of neuropsychiatric disorders. It will be key to determine how RmTBI received during key developmental periods affect susceptibility to neuropsychiatric disorders.

## Perspectives and conclusions

As we have mentioned above, the adolescent developmental period is a critical time in life that is characterized by increased risk-taking behaviors and increased risk for mTBI. Yet, adolescents are severely underrepresented in the mTBI literature. We believe the adolescent period is important for future research for a number of reasons. First, during adolescence, white matter tracts are not fully developed. As we have mentioned, in DAI white matter tracts are especially susceptible to axonal sheering and tearing. Does this mean adolescents will experience more severe injuries because the tracts have less tensile strength? Or will they be less likely to display white matter injuries? It is possible adolescents will be more vulnerable to this type of injury as they have increased water content in the brain and may be more disposed to cerebral edema [204]. This claim is supported by Cernak and colleagues utilizing a pediatric model of mTBI in Sprague-Dawley rats. Following a single injury temporal patterns of edema in the cortex and hippocampus differed in juvenile (P7, P14, P21) and adult animals whereby, juveniles displayed edema formation earlier than adults [205]. Given the immense changes to brain structure during adolescence, temporal profiles may not follow the same trajectory as pediatric injuries. Indeed, a single injury at P30 did not cause identifiable edema in adolescent mTBI. However, administration of a second injury exacerbated secondary cascades and increased edema [206].

The findings from Mannix (2017) provide important support for this concept: adolescents exposed to RmTBI displayed similar magnitudes of microgliosis to adults with RmTBI [40]. While this may seem unimportant, chronic microgliosis following RmTBI is associated with persistent behavioral differences in adult models [185]. While beyond the scope of this review, it is also important to note that astrocytes play important immune related roles following brain injuries, and chronic astrocytosis may additionally compound behavioral deficits (for in-depth reviews, see [207, 208]). Importantly, microglia and astrocytes are known to engage in cross talk whereby, activation of one, also activates the other, suggesting that both play vital roles in the pathophysiology and recovery from injury [209]. In addition, we have highlighted specific roles for microglia in synaptic pruning in this review and recent evidence suggests that astrocytes may also play roles in synaptic pruning [210]. The injury-induced activation of microglia during adolescence may disrupt homeostatic synaptic pruning and lead to decreased dendritic branching similar to what has been identified in the ageing or AD brain [173, 211]. Furthermore, both adolescence and TBI upregulate the complement cascade. Complement cascade expression is downregulated during non-developmental periods and is upregulated during adolescence. Kopec and associates confirmed that during adolescence, microglial-induced synaptic phagocytosis was mediated by protein C3 [109]. Further, C3 mRNA was significantly increased following experimental TBI [212]. Following RmTBI in adolescence, this upregulation of the complement cascade may mediate abnormal microglial synaptic pruning at otherwise strong, healthy synapses (Fig. 3) [164].

**Fig. 4:**
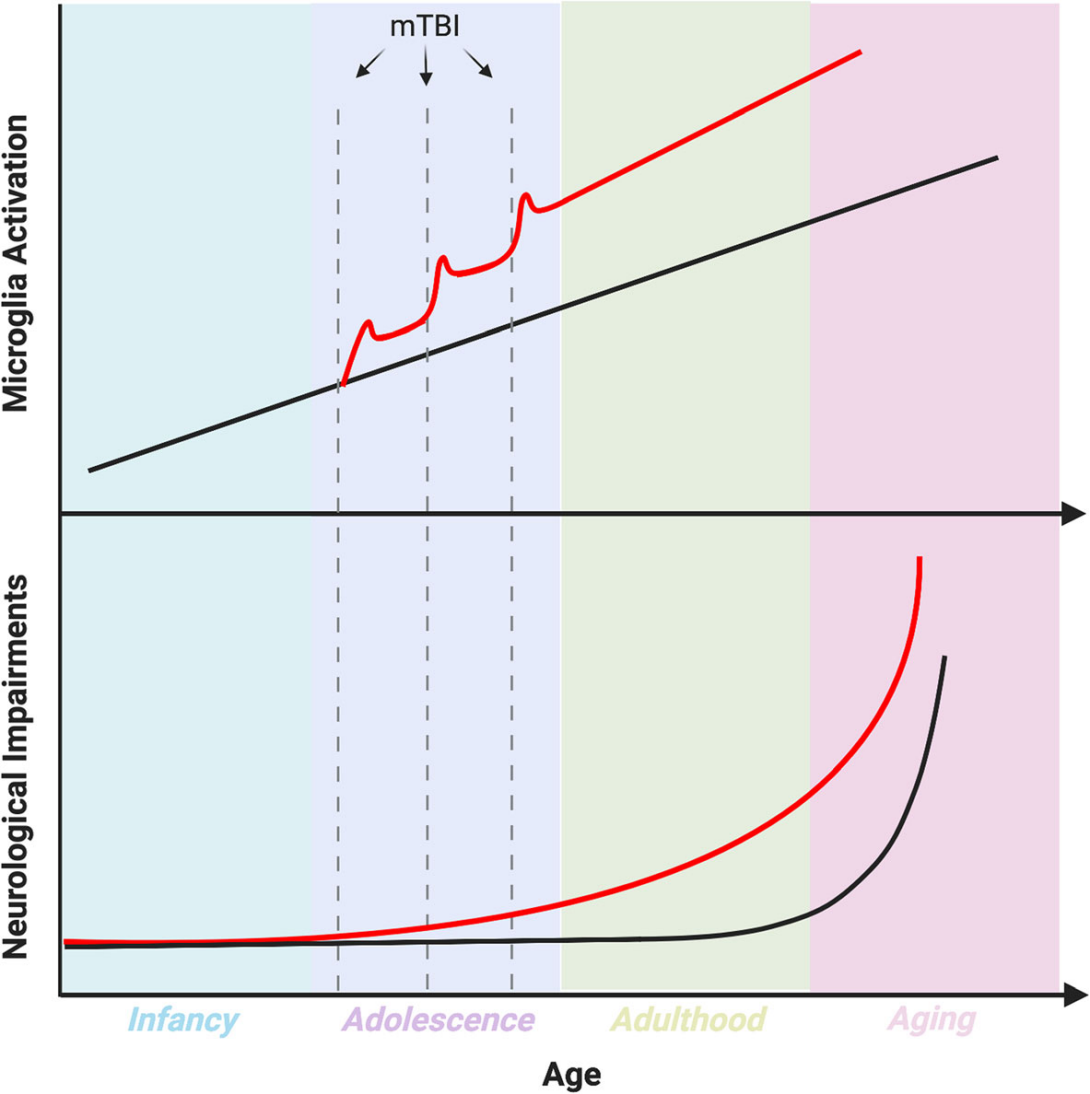
Trends in microglia activation and neurological impairments following adolescent RmTBI. Throughout life, microglia are known to become increasingly activated. In addition, neurological impairments increase throughout adulthood and ageing. RmTBIs sustained in adolescence may cause earlier chronic or primed microglia activation that may persist into adulthood and through aging. Increased microglia activation during the adolescent developmental period may therefore influence the acquisition or onset of neurological impairments throughout life. Disruption to neurological functioning may induce negative consequences through adulthood into aging

Finally, adolescent RmTBI may perpetuate abnormal microglia priming, reminiscent of aging where microglia lose the ability to shift from activated to ramified phenotypes. This is relevant because activated microglia are less capable of performing their homeostatic developmental functions. Studies have shown that microglial activation can persist up to 18 years following TBI in clinical populations [197]. It is therefore possible that adolescent RmTBI primes microglia to activated phenotypes earlier than usual, potentially leading to the early onset of neurodegenerative disorders and neurological decline (Fig. 4). While tracking adolescent RmTBI into adulthood has scarcely been researched, we do know that severe TBI in adolescence results in continuous verbal IQ decline and impairments in attention and working memory well into early adulthood [213]. Therefore, although adolescents are underrepresented in current TBI research, the literature to date suggests that receiving RmTBIs during adolescence may have far reaching consequences that require greater attention.

Research into mTBI and specifically RmTBI has seen a surge in recent years. However, the adolescent population has not been adequately studied and there is a specific void in the literature regarding the neuroinflammatory profile in this age group. Moreover, in the research that exists there is considerable variability and heterogeneity between studies. The main issues plaguing the mTBI research field are the lack of transparency and consistency in (1) model, (2) severity, (3) sex, (4) age and species, (5) time points for analysis, (6) number of injuries administered, and (7) inter-injury interval. For example, the inclusion of both males and females in basic and clinical research is of particular importance. Beginning in utero, and continuing throughout life, microglia development and transcriptional profiles differ between males and females. These differences in microglia phenotypes may manifest following injury and combine with sex-specific epigenetic changes, behavioral symptomologies, and microscopic structural changes to produce divergent pathological profiles. These differences are indicative of a non-uniform injury responses which have the potential to also impact therapeutic interventions and recovery. Given that the inflammatory profile differs based on each of the issues mentioned above and there is significant heterogeneity in the environmental factors surrounding injuries, it is currently difficult to draw specific conclusions regarding the role of microglia in adolescent and adult TBI pathophysiology. Notwithstanding, we have demonstrated that examination of the homeostatic and neurodevelopmental functions of microglia and immunological function during injury in adult states may provide novel insight into pathological consequences of activating these cells during adolescence. Sustaining injuries during this period has significant potential to alter developmental trajectories by augmenting microglial-dependent synaptic pruning, white matter tract development, and priming these cells to more activated phenotypes that could exacerbate neurological decline. The future however is bright. New technologies such as translocator protein (TPSO) autoradiography and positron emission technology (PET) have been developed to image microglia activation in vivo, in both humans [214–216] and rodents [217, 218]. These novel techniques open avenues to image microglia in living adolescents, which will be paramount to understanding this important aspect of neuroinflammation during such a critical period of brain development.

## Data Availability

Not applicable.
